# Controlled Growth Factor Delivery and Cyclic Stretch Induces a Smooth Muscle Cell-like Phenotype in Adipose-Derived Stem Cells

**DOI:** 10.3390/cells10113123

**Published:** 2021-11-11

**Authors:** Brandan Walters, Paul A. Turner, Bernd Rolauffs, Melanie L. Hart, Jan P. Stegemann

**Affiliations:** 1 Department of Biomedical Engineering, University of Michigan, 1107 Carl A. Gerstacker Building, 2200 Bonisteel Blvd, Ann Arbor, MI 48109, USA; bdw2116@gmail.com (B.W.); paultur@umich.edu (P.A.T.); 2 G.E.R.N. Center for Tissue Replacement, Regeneration & Neogenesis, Department of Orthopedics and Trauma Surgery, Faculty of Medicine, Albert-Ludwigs-University of Freiburg, Engesserstraße 4, 79108 Freiburg, Germany; bernd.rolauffs@uniklinik-freiburg.de

**Keywords:** adipose-derived stem cells, smooth muscle tissue engineering, microspheres, growth factors, cyclic stretch, cell morphology, cell shape, bladder, sphincter, vascular tissue engineering

## Abstract

Adipose-derived stem cells (ASCs) are an abundant and easily accessible multipotent stem cell source with potential application in smooth muscle regeneration strategies. In 3D collagen hydrogels, we investigated whether sustained release of growth factors (GF) PDGF-AB and TGF-β1 from GF-loaded microspheres could induce a smooth muscle cell (SMC) phenotype in ASCs, and if the addition of uniaxial cyclic stretch could enhance the differentiation level. This study demonstrated that the combination of cyclic stretch and GF release over time from loaded microspheres potentiated the differentiation of ASCs, as quantified by protein expression of early to late SMC differentiation markers (SMA, TGLN and smooth muscle MHC). The delivery of GFs via microspheres produced large ASCs with a spindle-shaped, elongated SMC-like morphology. Cyclic strain produced the largest, longest, and most spindle-shaped cells regardless of the presence or absence of growth factors or the growth factor delivery method. Protein expression and cell morphology data confirmed that the sustained release of GFs from GF-loaded microspheres can be used to promote the differentiation of ASCs into SMCs and that the addition of uniaxial cyclic stretch significantly enhances the differentiation level, as quantified by intermediate and late SMC markers and a SMC-like elongated cell morphology.

## 1. Introduction

As the population ages, higher numbers of patients will need to be treated for diseases characterized by dysfunction of smooth muscle, including cardiovascular disease [1], and urinary incontinence [2]. While technology offers many palliative options and changes in lifestyle can slow progression, tissue-engineered therapies offer one of the few potential options to reverse the degeneration of tissues by replacing or supplementing the tissues with alternative surrogates. In order to produce constructs to potentially treat affected tissues, a reliable and well-characterized source of smooth muscle cells (SMCs) is needed. Mesenchymal stromal cells (MSCs) offer a readily available source for these cells. In particular, adipose-derived stem cells (ASCs) are increasingly being considered for their higher yield during extraction, ease of access, and decreased donor co-morbidity [3,4,5,6]. Moreover, adipose-derived progenitor cells already express some smooth muscle markers [7,8,9,10], suggesting that ASCs may naturally (in vivo) contribute to muscle development. ASCs were first recognized for their adipogenic properties [11,12,13,14,15,16] but have since also been demonstrated in vitro to be osteogenic [11,12,13,14,16,17] and chondrogenic [13,14,18,19]. More recently, their potential as a source for generating SMCs has attracted considerable interest [13,20,21,22], but substantial work is needed to better control the differentiation of ASCs into a SMC lineage and to determine the best method to induce this process.

The extracellular environment of SMCs and their progenitors contains a range of stimuli that induce and maintain their phenotype. Growth factors have been actively used to recapitulate some of these stimuli and to induce a SMC phenotype from other cell lineages. The transforming growth factor beta (TGF-β) superfamily of cytokines was first discovered in cancer cell lines and was noted for the ability to fundamentally change the phenotype of fibroblasts [23]. TGF-β1 in particular has been noted for its ability to induce differentiation of MSCs into both chondrocytes [24,25] and SMCs [26,27,28,29,30]. Other growth factors that that have been shown to induce SMC differentiation are platelet-derived growth factor (PDGF) [28,29,31], transforming growth factor β3 (TGF-β3) [32], sphingosylphosphorylcholine (SPC) [32], and bone morphogenic factor 4 (BMP4) [33,34,35]. These growth factors have been shown to, at least transiently, upregulate the expression of SMC-specific genes and proteins [26,27,28,29,30,32,33,35]. They are typically delivered to in vitro cultures by dissolving and continuously adding them in culture media. However, replenishing growth factors in vivo would be more difficult, especially when bolus injections are diluted into surrounding tissues, which may impede their function. To ensure sufficient GF concentrations over time, several groups have developed methods to load growth factors into the cell substrate [36] or into modular delivery devices [37,38] in an effort to promote local and sustained delivery [24,38]. These methods have been used to deliver bone morphogenic protein 2 (BMP2) [25,37,38], vasculogenic endothelial growth factor (VEGF) [38], fibroblast growth factor (FGF) [39], and TGF-β1 [24,25,40]. Because of their size and general shape, these carriers have been called microspheres (μspheres) and they hold potential to augment growth factor delivery for clinical uses.

Another commonly used method for inducing a SMC-like phenotype in progenitor cells is mechanical stimulation. As an example, this stimulation is necessary for bladder development, since mechanical stretch regulates the in vitro survival of human bladder SMCs [41]. Cyclic strain has been used to induce MSC types [42,43,44,45,46], including ASCs [20,30,47], into a SMC-like phenotype [30,43,44,47]. Additionally, cyclic strain has been shown to induce the alignment of a variety of cell types. For example, in cells seeded on 2D silicone or non-fibrous substrates, cyclic strain caused cells to perpendicularly align [20,48,49,50], while cells seeded on collagen and other fiber-based scaffolds aligned parallel to the strain direction regardless of culture dimensionality [51]. These findings led our group to suggest that MSC alignment in response to cyclic stretch depends on the properties of the substrate, and that alignment plays an instrumental role in generating specific cell morphologies through cyclic stretch [52]. Cyclic stretch can be used alone [46] or in combination with growth factors, which maximizes the effects on cell phenotype [20,30].

Regardless of culture dimensionality or the presence of growth factors, stem cells exhibit a change in morphology in response to mechanical stimulation. In fact, it has been found that growth factors and stretch affect both the cell size [19,25,32,33,40,48,49] and the polarization [20,26,47,50,53] of cells as they differentiate, particularly in mesodermal lineages. Fabrication methods that allow the printing of specific geometries and areas of cell adhesion sites have been informative in understanding how cells regulate differentiation and offer an approach to direct this process [13,54,55]. Controlling cell spreading by altering ligand presentation [56] or limiting elongation [57,58] offers another means of regulating differentiation. In this context, cell shape has proven a valuable parameter in predicting and directing phenotype [59,60] with many groups using it as a qualitative indication of the differentiated state.

Previously, our group has used the relationship between cell shape and phenotype on 2D substrates to quantify changes in cell morphology throughout the process of bone marrow-derived MSC differentiation towards SMC-like cells [61]. These 2D studies showed that addition of cyclic strain or the addition of growth factors is capable of inducing differentiation of MSCs into a SMC-like phenotype. 3D collagen environments have been suggested to dedifferentiate SMCs but biochemical and mechanical stimulation have been shown to recover this lost expression [62]. We theorized that these two stimuli, well documented to induce SMC differentiation, could also promote the differentiation process in these 3D structures and enhance the properties of the tissue-engineered constructs. In this context, the present study examined the differentiation of ASCs into SMC-like cells in 3D collagen hydrogels in response to (i) the sustained release of PDGF-AB and TFG-β1 from growth-factor-loaded μspheres, (ii) uniaxial cyclic stretch, or (iii) a combination of growth-factor-loaded μspheres with cyclic stretch. To this end, we investigated the individual and combined effects of cyclic strain and controlled growth factor delivery by (i) quantifying the resulting cell morphology using a panel of shape descriptors [52,61] to capture the full range of morphological changes and (ii) characterizing the expression of early and late SMC markers on the protein level. Understanding the effects of these differentiation stimuli on SMC marker morphology and expression would not only improve our technical ability to generate SMCs, but will also help confirm the quantitative aspects of cell morphology that can be used as ASC differentiation markers. In turn, these insights may lead to new standard protocols and improved methods for inducing and characterizing cell phenotype in regenerative medicine.

## 2. Materials and Methods

### 2.1. ASCs Culture and Expansion

Human adipose-derived mesenchymal stem cells isolated from adult normal, healthy subcutaneous adipose tissue (Rooster Bio, Frederick, MD, USA, Catalog #MSC-021) were expanded in T175 flasks in Alpha Modification Essential Medium (Alpha MEM, Fisher, Rockville, MD, USA) supplemented with 10% qualified fetal bovine serum (FBS; Invitrogen, Carlsbad, CA, USA) and 1% penicillin and streptomyocin (PS; Invitrogen). Cells were cultured at 37 °C and 5% CO_2_ and their media was changed every three days. Cells were passaged at 70–90% confluency and passages 5–7 were used for experiments. On day 0, when ready to be used for producing constructs, cells were washed in PBS and incubated in 0.25% Trypsin (Fisher) at 37 °C for 3–4 min to lift the cells from the flasks. After cells had lifted, they were counted, centrifuged, and resuspended in Dulbecco’s Modified Essential Media (DMEM, Fisher) for use in collagen hydrogels. This DMEM was also supplemented with 10% qualified FBS (Invitrogen) and 1% penicillin and streptomyocin (Invitrogen).

### 2.2. Production of μSpheres

A 10% by weight gelatin A stock solution was made by dissolving gelatin A (Sigma G6144, St. Louis, MO, USA) in a 2.5% *v/v* NaOH solution and vortexing vigorously which was then incubated at 37 °C until completely dissolved. A 6% working solution was produced by diluting the 10% stock gelatin with water. A 1% genipin crosslinker was made by dissolving genipin (Wako 078-3021, Fisher) in PBS which was heated and mixed until dissolved into a yellow color. The genipin stock solution was kept at 4 °C until use. A0.01% PBS + L101 oil washing solution was dissolved by adding Pluronic L101 (BASF, Ludwigshafen, Germany) to PBS at 4 °C and was stored at room temperature until use. For production of μspheres, 100 CS silicone oil (Clearco, Willow Grove, PA, USA) was added to a beaker and warmed to 37 °C. The working 6% gelatin solution was also warmed to 37 °C. The impeller of a Servodyne mixer (Cole Palmer, Vernon Hills, IL, USA) was immersed into the beaker containing the silicone oil. A speed of 2300 RPM was set to ensure lack of splashing. Slowly the desired volume of 6% gelatin was added to the beaker and the gelatin was allowed to emulsify for 5 min. The rotor was placed on a 66% duty cycle which ran every 1.5 min for 3 min. The silicone and emulsified gelatin was then placed on ice and mixer was allowed to run for another 30 min. 25 mL of the gelatin oil mixture was then added to 50 mL tubes and diluted 1:2 with a PBS + L101 solution which was mixed for 5 min by constantly inverting the tubes on a Rotoflex tube rotator (Argos, Vernon Hills, IL, USA). The tubes were then centrifuged at 300× *g* for 5 min and the top oil phase was removed. The bottom phase was transferred to new tubes and the washing step was repeated by bringing each tube’s volume to 50 mL again with PBS + L101. The tubes were once again centrifuged and the oil phase removed. Repeated washes were performed until the remaining gelatin and PBS phase was approximately 10–15 mL. This was then vortexed and 1% genipin was added. The tubes were allowed to mix overnight (18–24 h) on the Rotoflex tube rotator allowing genipin to crosslink. The next day, tubes were centrifuged at 200× *g* for 5 min. The liquid phase was removed and the volume was adjusted to 50 mL by adding ethanol (200 proof, 459,844 Sigma). The tubes were then inverted on a Rotoflex rotor for 1 h, centrifuged at 200× *g* for 5 min and washed twice more with ethanol and then three times with water. Sonified gelatin μsphere using 50% duty cycle every minute for 3 min with Digital 250 Sonifier (Branson, Danbury, CT, USA). Gelatin μspheres were freeze dried until use.

### 2.3. Loading Growth Factor into μSpheres

Lyophilized μspheres were massed into three sterile centrifuge tubes. The first contained 0.11 mg of gelatin μspheres. The second contained 0.27 mg of μspheres. The third contained 0.38 mg of μspheres. Tubes were centrifuged to collect the μspheres at the bottom. TGF-β1 (20 μg/mL PBS, Peprotech, Rocky Hill, NJ, USA), PDGF-AB (20 μg/mL (100-00AB-10UG, Peprotech, Rocky Hill, NJ, USA), or PBS was then added to the first, second and third tubes, respectively, at 10 μL/mg of μspheres. Each tube was then centrifuged to collect the growth factor and μspheres at the bottom and incubated overnight at 37 °C. The μspheres were resuspended with FBS (Invitrogen) to a final concentration of 10 mg μspheres/mLand incubated at room temperature for one hour before homogenization using a Digital Sonifier (Branson) set at 10% amplitude for 2 min with a 50% duty cycle every 20 s.

### 2.4. Tissue Construct Production and Culture

**Figure 1:**
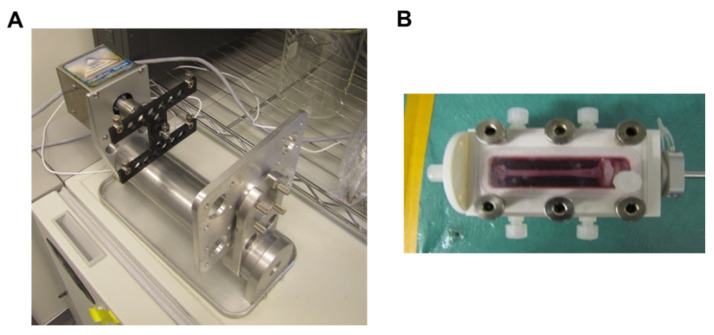
Image of the uniaxial cyclic stretch bioreactor. (**A**). The frame and motor showing the rack that holds four separate bioreactor chambers. (**B**). A single bioreactor chamber showing the construct mounted in the medium chamber.

Type I collagen (MP Biomedicals, Solon, Ohio) was dissolved in 0.02 M acetic acid (Sigma) to a concentration of 4 mg/mL. Collagen gels were made according to previous studies [63]. Briefly, to make 2 mg/mL gels, 4 mg/mL collagen composing 50% of the final gel volume was added to different tubes, one for each type of growth factor loading. To each tube, a volume of 5× DMEM (Fisher), equal to 20% of the final gel volume, and 0.01 M NaOH (Sigma), equal to 10% of the final volume, was mixed with the collagen to neutralize acid and to make the solution isotonic. FBS (Fisher) equal to 10% of the final volume was added with the respective amounts of μspheres to each tube. For controls gels (Control), no μspheres and no growth factors were added to the FBS. For gels with unloaded μspheres and growth factors in the media (GF), 38% of the FBS contained control μspheres (10 mg of μspheres/mL FBS). For gels with growth factor loaded into the μspheres (LS), 11% of the FBS contained μspheres TGF-β1 and 27% of the FBS contained μspheres loaded with PDGF-AB (both at 10 mg μspheres/mL FBS). ASCs, which were suspended at 5e^6^ cells/mL in 1× DMEM (Fisher), were mixed in, equal to 10% of the final gel volume. The hydrogel mixtures were then dispersed to wells for static culture or bioreactor chambers for mechanical stimulation. 0.5 mL of the hydrogel solution containing the cells was dispersed to wells of a 24 well TC-treated plate (Corning, Fisher) and 4 mL was dispensed to each bioreactor chamber custom made for stretching (TGT LigaGenTM, Minnetonka, MN, USA) (Figure 1). All plates and chambers were incubated and allowed to equilibrate to 37 °C. After the gels set, 0.5 mL of respective media was added to each static gel and 4 mL of respective media was added to each construct in the bioreactor chambers. Control gels (Control) received 1× DMEM with 10% FBS (Fisher), gels with unloaded μspheres (GF US) received 1× DMEM with 10% FBS, 5 ng/mL PDGF (Peprotech), 5 ng/mL TGF-β1 (Peprotech), and 30 μM ascorbic acid, and gels containing loaded μspheres received 1× DMEM with 10% FBS and 30 μM ascorbic acid. Constructs were incubated at 37 °C until each gel’s respective media was changed on day 3 (D3). Gels in bioreactor chambers were released from their molds and suspended from anchor points before replacing the media. On D4 samples were collected and analyzed while all remaining gels continued to be cultured. The gels in bioreactor chambers were stretched starting on D4 (described below). Media was changed again on D6 and the final samples were collected for analysis on D7.

### 2.5. Determining Growth Factor Release

PDGF μspheres and TGF-β1 μspheres (Loaded) were incubated in 5.0 U/mL collagenase I (MP Biomedical) for 24 h. Empty μspheres in collagenase served as negative controls (Control) and growth factor, equal to the amount loaded into the μspheres, was dissolved in PBS to serve as a positive control (GF in PBS). Samples from the PDGF μspheres and controls were collected at 0, 1, 2, 3, 4, 7, 9, 11, 14, and 24 h. Samples from the TGF-β1 μspheres and controls were collected at 0, 4, 7, 9, 11, 14, and 24 h. Samples were analyzed using a PDGF-AB Quantikine ELISA Kit (DHD00C, R&D Systems) and Human TGF-β1 Quantikine ELISA Kit (DB100B, R&D Systems). Samples were analyzed on a Synergy H1 microplate reader (BioTek, Winooski, VT, USA), accounting for the removed volume at each time point. The time point that growth factor release plateaued was recorded. Control μspheres were kept in 4.0 and 5.0 U/mL collagenase I (MP Biomedical) and samples were collected every 15 min until the μspheres were fully dissolved (~12 h). Exploiting the autofluorescence of the genipin-crosslinked gelatin, a Synergy H1 microplate reader (BioTek) was used to measure the amount of gelatin dissolved in each sample. This was calculated by normalizing the fluorescence by that of fully dissolved μspheres and, again, accounting for the volume removed during each sampling. The percentage of μsphere degradation that occurred when growth factor release plateaued was recorded. ASCs in collagen hydrogels with control μspheres as described above were cultured for two weeks collecting media samples every two days. Using the autofluorescence of the solubilized genipin-crosslinked gelatin, the amount of degradation was calculated accounting for volume removed from each sample. The time it would take ASCs to degrade the μspheres and facilitate the highest amount of release was calculated using the degree of degradation known to correspond to full release in collagenase. To examine the effects of growth factor delivery method on the degradation of the μspheres, ASCs were cultured with loaded μspheres (LS as described above), with unloaded μspheres and growth factor in the media (GF as described above), and control conditions (Con as described above) for one week changing respective media on D3 and D6. Removed media was analyzed for solubilized gelatin with the Synergy H1 microplate reader.

### 2.6. Stretching Gels

As described above, 4 mL hydrogels from each growth factor delivery condition (LS, GF, and Con) were cast into custom molds inside bioreactor chambers designed to facilitate cyclic uniaxial stretch (TGT LigaGenTM). On D3, during the standard media change, the molds for setting the hydrogels were removed and constructs were suspended in fresh media between two anchoring points. On D4 the chambers with hydrogels were inserted in a bioreactor (TGT LigaGenTM) and proprietary software was used to apply cyclic uniaxial stretch at 10% strain and 1 Hz for 6 h a day for 3 consecutive days (D4, D5, D6). On D6 after stretch, media from each chamber was replaced with the media for that respective condition in parallel with the respective control gels. On D7, samples from each chamber were collected and analyzed for shape, gene expression, and protein expression.

### 2.7. Fluorescence Microscopy for Cell Shape

The ASCs-seeded constructs were collected and stained on D4 and D7 using Calcein (1:1000, Lifetech/Fisher) and diamidino-2-phenylindole (DAPI, 1:1000, Lifetech, Fisher) based on our previous work [46,52,61]. ASCs embedded in collagen gels were imaged using an Olympus IX15 Microscope system (Olympus America, Center Valley, PA, USA). Calcein-stained ASC morphology was detected using the green filter and the nuclei were imaged using a blue filter. The individual shapes of large quantities of ASCs were measured simultaneously through 2D projections of fluorescence with an ImageJ macro (National Institute of Health, Bethesda, MD), according to [61]. The following six mathematical shape descriptors were quantified for each MSC: length (major axis), area, circularity (4*π(area/perimeter^2), projection factor (perimeter^2/area), roundness (4*area/(π*major axis length^2)), and aspect ratio (major axis angle/minor axis). The aspect ratio is the ratio of the cells width to its height. Projection factor represents a cell enlargement with irregular boundaries or protrusions.

### 2.8. Compaction of Static Constructs

Every day and before collection of samples for analysis, images were taken of each gel using a bright field microscope (Olympus). In ImageJ (NIH), the long and short access of each sample were measured and approximated as an ellipse. The areas of the resulting ellipses were normalized to their initial area of the gels, effectively, the area of the well.

### 2.9. Immunocytochemistry and Analysis of Samples

Constructs were collected on D7, washed twice with PBS, and fixed with Z-fix (Anatech, Battle Creek, MI, USA) for 10 min at 4 °C. Samples were washed twice with PBS and permeabilized with 0.5% Triton X-100 (Sigma) for 20 min at room temperature. All samples were washed twice more in PBS and kept at 4 °C until staining. When staining, primary antibodies against smooth muscle actin (SMA, Millipore: ABT1487, Burlington, MA), transgelin protein (SM22, Santa Cruz: 50446, Dallas, TX, USA), and smooth muscle myosin heavy chain (MHC, Millipore: MAB3568) we dissolved in 10 mg BSA. Samples were incubated at room temperature in primary antibodies for 2 h at 1:200, 1:50, and 1:50 for anti-SMA, anti-SM22, and anti-MHC, respectively. Samples were washed twice in PBS and incubated in secondary antibody for 1 h at room temperature. 488 Alex Fluor anti-rabbit (1:200, ThermoFisher, A-11070) or 488 Alexa Fluor anti-mouse (1:250, ThermoFisher, A-11001) were used as secondary antibodies to for detection of SMA and SM22 or MHC, respectively. DAPI (1:1000, Lifetech) was added to the secondary stain as well. Secondary antibodies and DAPI were washed off twice with PBS in low light settings. Samples were stored at 4 °C until imaging. Imaging was done on a Nikon A-1 Spectral Confocal Microscope (Melville, NY) maintaining exposure levels across all samples when imaging specific proteins. Analysis of images were carried out in ImageJ. Pixel intensities were summed across an image to obtain a “raw integrated intensity” value for each image. This value was normalized to the number of nuclei in the image to account for regional differences in the number of cells.

### 2.10. Statistical Analysis

All data for bar graphs were plotted and statistically analyzed in R. ANOVA and Dunn’s Method were used for post hoc analyses to compare individual groups. For bar graphs comparing different GF treatments on shape or gene expression, only significant differences between conditions at the same time point were indicated using a bar above the two conditions. Differences between growth factor treatments from different days were not indicated, regardless of significance. For bar graphs comparing the effects of stretch on shape or gene expression, only statistical differences between stretched and static controls were indicated (blue ‘a’ for Control, red ’b’ for GF, and a green ‘c’ for LS). When comparing differences in proteins expression between different growth factor delivery treatments or comparing the effects of stretch, a letter was used to comparing static and stretched conditions (blue ‘a’ for Control, red ’b’ for GF, and a green ‘c’ for LS) and a bar above conditions indicates statistical difference between growth factor treatment within the same mechanical loading treatment. Correlation analyses were performed with SigmaStat (Systat Software, Version 12.5, San Jose, CA, USA), using the Pearson product moment correlation test. All error bars are plus/minus the standard error of the mean.

## 3. Results

### 3.1. Schematical Outline of Experimental Setup

**Figure 2:**
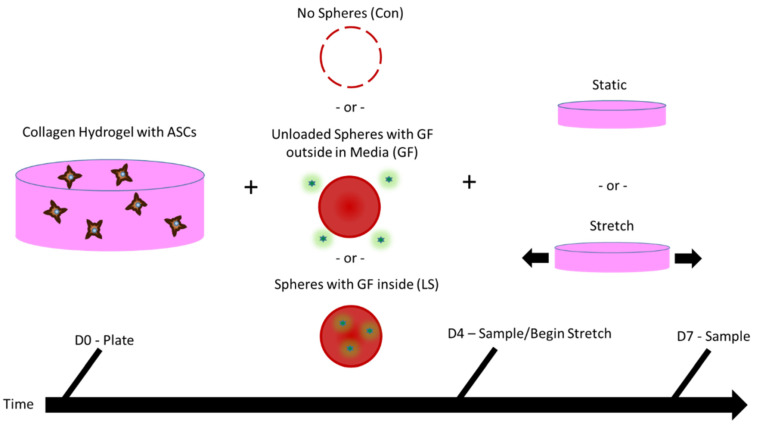
Methods for ASC construct production, culture, and sampling. Adipose-derived stem cells (ASCs) were expanded and then seeded into collagen hydrogels. The hydrogels contained either no µspheres and no growth factors (Control), unloaded control µspheres with growth factors in the medium (GF), or µspheres loaded with growth factors (LS). For four days, the gels were cultured either in media containing no additional growth factor (Control), media containing growth factors and ascorbic acid (GF; US, 5 ng/mL PDGF, 5 ng/mL TGF-β1, and 30 μM ascorbic acid), or media containing just ascorbic acid (LS; 30 μM ascorbic acid). On day 4 (D4), a subset of samples was collected for analyses. A subset of the remaining samples were stretched in a bioreactor once a day for three days (10% strain at 1 Hz for 6 h), whereas another subset was statically cultured during this time. On day 7 (D7), all remaining samples were collected for analyses.

ASCs were seeded in 3D into collagen hydrogels along with (i) either no μspheres and no growth factors (Control), (ii) unloaded μspheres with growth factors in the media (GF), or (iii) with μspheres that had been loaded with growth factors (LS). At day 4 (D4) of culture, a subset of the samples was collected for analyses. The remaining samples were either statically cultured for an additional three days and analyzed on day 7 (D7), or were uniaxially stretched for 3 days from D4 to D6, as described in the Methods. On D7, all remaining samples were collected for analyses. This process is outlined schematically in Figure 2. Additional samples were used for analyzing hydrogel compaction.

### 3.2. Growth Factor Release from Genipin-Crosslinked Gelatin μSpheres

**Figure 3:**
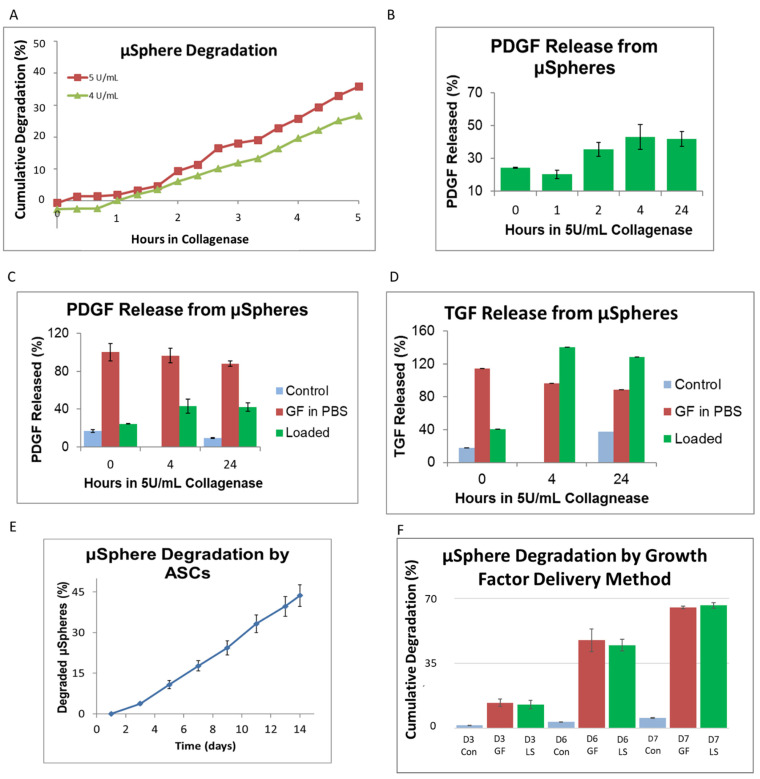
Growth factor release from μspheres. When control spheres were kept in collagenase I, they degraded at a linear rate dependent on enzyme concentration ((**A**), *n* = 5 for each time point). ELISAs were used to compare the release of PDGF ((**B**,**C**), *n* = 3 for each time point and concentration) or TGF-β1 ((**D**), *n* = 3 for each time point and concentration) from loaded μspheres (Loaded) being degraded in Collagenase. μSpheres with PDGF-AB (Loaded) showed gradual release from μspheres up to 4 h where it plateaued with a similar amount released 4 h and at 24 h (**B**). We compared this release against positive controls where PBS was loaded with the same absolute amount of protein as in the μspheres and using the same concentration of collagenase (GF in PBS). An empty negative control contained empty μspheres and collagenase (Control). The positive controls (Growth Factors in PBS) remained relatively constant, around 100%, throughout all of the time points and the negative control (Control) remained at baseline levels from the beginning to the end of the time course (**C**,**D**). μSpheres with PDGF (**C**) and TGF-β1 (**D**) initially showed baseline level concentrations of growth factor comparable to negative controls (Control) but by 4 h the highest amount of each growth factor had been released. This level was maintained throughout 24 h of collagenase incubation (**C**,**D**). For PDGF this was approximately 45% of the amount loaded into the spheres (**C**) while TGF-β1 levels were higher than positive controls (**D**). When control μspheres were cultured with ASCs in hydrogels (*n* = 4 for each time point), degradation was linear and it took approximately 10 days to degrade 30% of the spheres (**E**). When growth factors were present either within the spheres (LS) or in the surrounding media (GF), ASCs were degraded over 35% by D6 but there were no differences in the levels of degradation of spheres between the two growth factor delivery methods ((**F**), *n* = 5 for each condition and time point).

To estimate the delivery rate of growth factors, degradation and delivery studies were first performed on the microspheres, as shown in Figure 3. Collagenase facilitated an approximately linear degradation of gelatin μspheres with 5.0 U/mL degrading approximately 7% of the μsphere mass per hour and 4.0 U/mL degrading approximately 5% of the mass per hour (Figure 3A). Loaded μspheres containing PDGF-AB were digested using 5.0 U/mL and GF release was gradual before plateauing around 4 h (Figure 3B). With μspheres left undigested, PDGF-AB release was similar to that of empty μspheres at 0 h and reached around 50% of the positive control (growth factor in media) release by 4 h of degradation (Figure 3C). This implied that maximum GF release occurred when ~30% of the μspheres had been digested, as seen by comparing the degradation profile (Figure 3) with the analogous GF release profile (Figure 3C). Corresponding experiments were performed on μspheres containing TGF-β1 and again, the immediate release of TGF-β1 from loaded μspheres at time 0 was comparable to negative controls. By 4 h in collagenase, the release of TGF-β1 had plateaued (Figure 3D). Like PDGF-AB, it appeared that by the time 30% of the μspheres had been degraded, all TGF-β1 had been released into solution.

Knowing the relative release profile of growth factors from μspheres, we next assessed how cells would degrade the μspheres and thereby release growth factors. When ASCs were cultured with control unloaded μspheres, the degradation rate was nearly linear and they degraded approximately 3% of the μsphere mass per day (Figure 3E). This suggests that by D10, ASCs should degrade μspheres to a degree that results in the maximum release of growth factors. To test if the presence of growth factors would affect cell-induced degradation of the μspheres, ASCs were cultured with unloaded μspheres (with the growth factors in the media, GF) or with growth-factor-loaded μspheres (LS) and the mass of μspheres degraded was measured over time. Regardless of how the cells were cultured with the growth factors, either through media or loaded μspheres, the presence of growth factors accelerated degradation to around 6% of the mass per day (Figure 3F). This implied that cells would degrade enough of the μspheres by D7 to facilitate optimal release of the growth factors.

### 3.3. Compaction of Constructs Cultured with Specific Growth Factor Treatments

**Figure 4:**
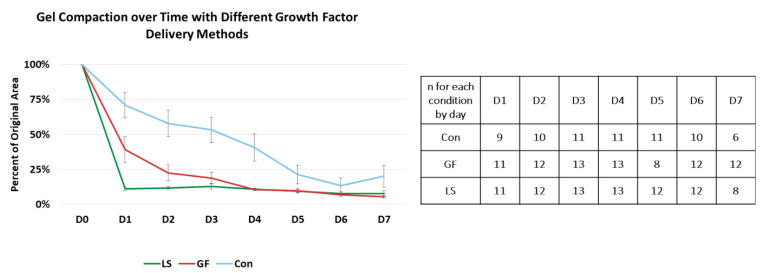
Compaction of gels with different growth factor delivery treatments. The blue line represents control gels with no growth factors, the red line represents gels with unloaded μspheres and growth factor in the media, and green line represents gels with growth factors in loaded μspheres. The table displays the number of samples measured from each condition at each time point. By D1, constructs with growth factors loaded into the μspheres (LS) were more compacted than constructs with growth factor in the media (GF) which were more compacted than gels without any treatment (Control) (*p* < 0.05). By D2 and through D5, LS and GF were statistically similar but they were both still statistically more compacted than the Con. By D6, all constructs had compacted to approximately 10% of their original area.

All constructs started compacting after being placed in culture, as shown in Figure 4. By Day 1, hydrogels with growth-factor-loaded μspheres (LS) had compacted to approximately 10% of their original volume, while hydrogels with growth factor in the media (GF) had compacted to approximately 40% of their original volume. In contrast, control hydrogels without growth factors had only compacted to approximately 70% of their original volume. Starting on D2, both delivery methods of growth factor induced statistically similar compaction, but both growth factor-containing treatments were more compacted than the control hydrogels. The control hydrogels continued to compact until they were statistically similar to the growth factor-treated hydrogels on D5. By D6, all hydrogels had compacted to approximately 10% of their original area, indicating that the choice of growth factor delivery method had impacted the rate of hydrogel compaction but not the final degree of compaction.

### 3.4. Morphology of ASCs Differentiated Using Growth Factor Treatments and Mechanical Stretch

**Figure 5:**
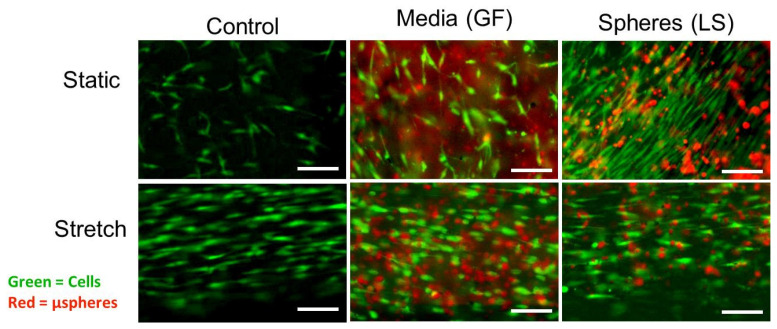
Morphology of ASCs cultured with different mechanisms of growth factor delivery in either static or mechanically stretched culture (D7). Green indicates live ASCs stained with Calcein; red indicates μspheres (unloaded and loaded). ASCs were cultured with either no μspheres (Control), with unloaded μspheres but containing growth factor in the media (GF), or with μspheres preloaded with PDGF and TGF-β1 (LS). A subset of these hydrogels was cyclically strained for 6 h a day for 3 days starting on D4. Scale bar represents 50 μm.

On D7, ASCs cultured in static conditions with growth factors, especially in the presence of loaded μspheres, appeared more spindle or rod shaped than control cells, as shown in Figure 5. In particular, cells immediately adjacent to growth-factor-loaded μspheres exhibited SMC-like morphology. After cyclic strain, regardless of the growth factor treatment, all cells had aligned with the axis of stretch (the horizontal direction in the images in Figure 5). Stretched cells had fewer lateral projections and were more elongated along the axis of stretch. Differences between the growth factor treatments in stretched cells were less obvious. These observations were quantified and compared with results presented in subsequent sections.

### 3.5. The Shape of ASCs with Induced SMC Differentiation Using Growth Factor Treatments

**Figure 6:**
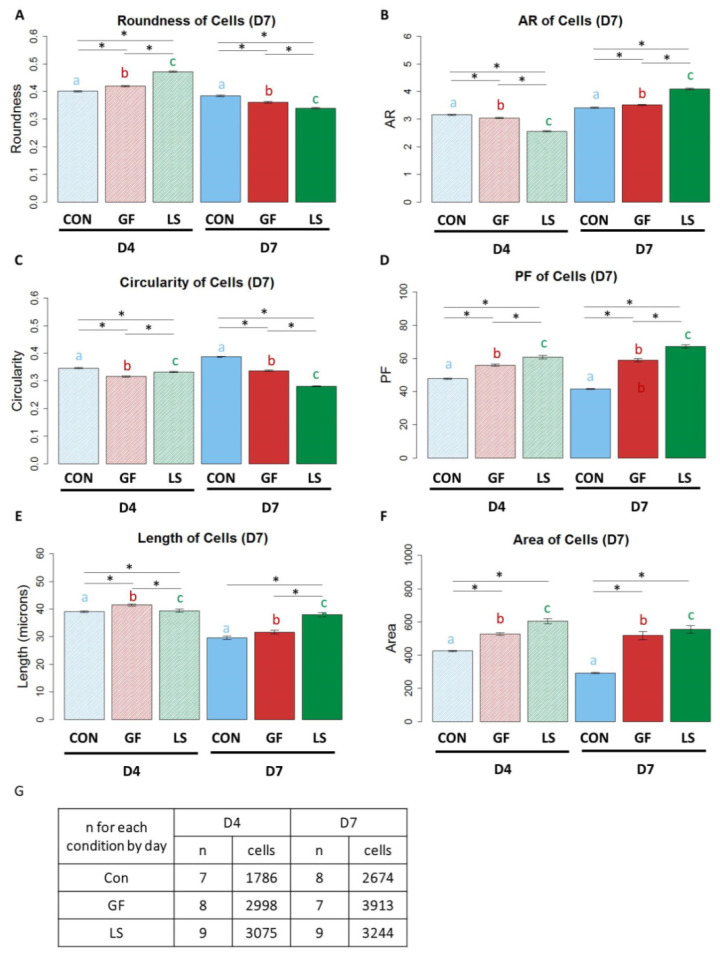
The shape of ASCs differentiated with different growth factor delivery methods. (**A**–**G**) Blue bars represent control gels with no growth factors (Con), red bars represent gels with unloaded μspheres and growth factor in the media (GF), and green bars represent gels with growth factors in loaded μspheres (LS). Bars with striated patterns represent samples from D4 and solid bars with fuller tones represent samples from D7. Lines ending above conditions with an asterisk (*) above indicate statistical differences between cells with different groups (*p* < 0.05). Letters above bars indicate significant differences between D4 and D7 data for CON vs. CON (as indicated by the letter a in blue), GF vs. GF (as indicated by the letter b in red), LS vs. LS (as indicated by the letter c in green) (*p* < 0.05). Panel G indicates the number of biological replicates and the numbers of cells from each condition.

Quantification of ASC morphology after treatment with growth factors is shown in Figure 6. On D7, the delivery of growth factors via μspheres (LS) produced large cells with a spindle-shaped, elongated SMC-like morphology. ASCs treated with growth-factor-loaded μspheres (LS) exhibited the highest roundness on D4, followed by ASCs with unloaded μspheres and growth factors in the media (GF), while control-treated ASCs were the least round. By D7, the control ASCs were the most round cells and LS ASCs were the least round (Figure 6A, *p* < 0.05). The aspect ratio revealed a similar trend of change in morphology; control ASCs had the highest aspect ratios, followed by GF ASCs and then LS ASCs on D4. However, by D7, the LS ASCs had the highest aspect ratio (Figure 6B, *p* < 0.05), indicating that over time the LS-treated ASCs generated the most spindle-shaped cells. On D4, GF ASCs had the lowest circularity followed by LS ASCs and then control ASCs. By D7, LS ASCs showed the lowest circularity (Figure 6C, *p* < 0.05). LS ASCs had also the highest projection factor values, followed by GF ASCs and then control ASCs on both D4 and D7 (Figure 6D, *p* < 0.05). Initially, on D4, LS ASCs were the shortest but by D7, LS ASCs were significantly longer than control ASCs and GF ASCs (Figure 6E, *p* < 0.05). GF ASCs and LS ASCs had larger surface areas than control ASCs at both time points (Figure 6, *p* < 0.05).

Moreover, Figure 6 highlights that treatment can affect cell shape differently after day 4 and day 7. For example, in the static LS group roundness is increased in the presence of loaded spheres after 4 days but is decreased after 7 days. These differential changes can be explained by the fact that the cell shapes at day 7 result from effects of the chosen experimental conditions as well as the effect of time. We noted such effects of time in our previous study [61] and confirmed time-dependent effects in the present study as well since all investigated day 4 vs. day 7 shape descriptors of a given experimental group were significantly different from each other. Therefore, cell shape is time sensitive.

### 3.6. Shape of ASCs Cultured with Growth Factor Treatments and Mechanical Stretch

**Figure 7:**
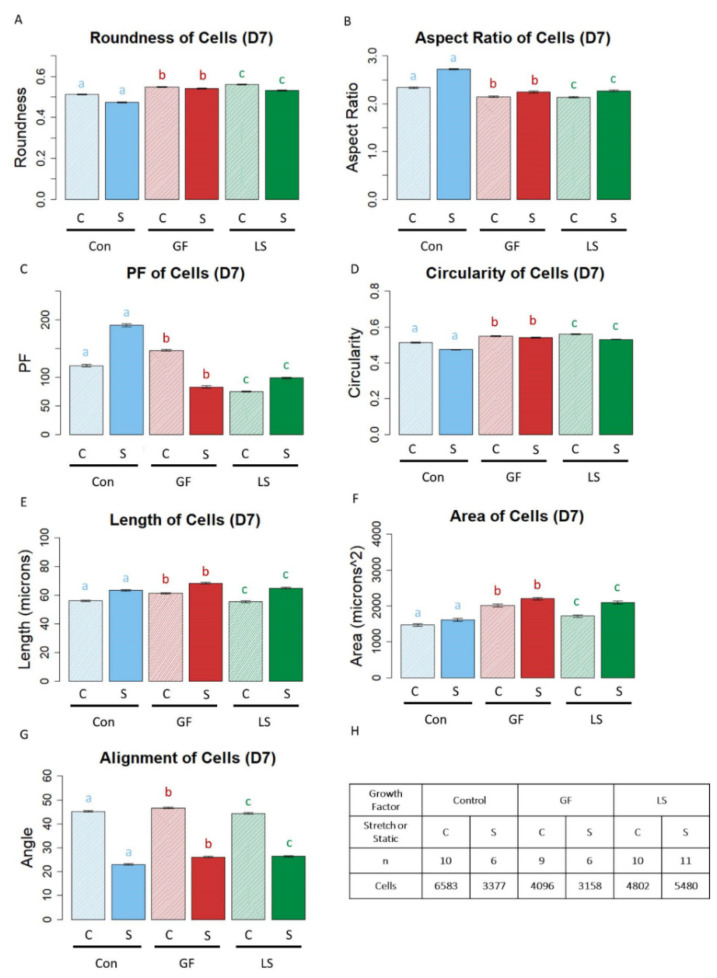
The Shape of ASCs in mechanically stretched constructs compared to statically cultured constructs. (**A**–**G**) Blue bars represent control gels with no growth factors (Con), red bars represent gels with unloaded μspheres and growth factor in the media (GF), and green bars represent gels with growth factors in loaded μspheres (LS). Bars with striated pattern represent unstretched controls and solid bars with fuller tones represent stretched samples. Lines ending with an asterisk (*) above indicate statistical differences between cells with different groups (*p* < 0.05). Letters above bars indicate significant differences between D4 and D7 data for CON vs. CON (as indicated by the letter a in blue), GF vs. GF (as indicated by the letter b in red), LS vs. LS (as indicated by the letter c in green) (*p* < 0.05). Panel H indicates the number of biological replicates and the numbers of cells from each condition.

In corroboration of the qualitative cell shapes presented in Figure 5, quantitative assessment of morphology revealed that cyclic strain had produced the largest, longest, and most spindle-shaped cells regardless of the presence or absence of growth factors or the growth factor delivery method. More specifically, ASCs in stretched constructs were less round than their statically-cultured counterparts, regardless of the growth factor delivery treatment (Figure 7A, *p* < 0.05). Subsequently, stretched cells had higher aspect ratios than static ASCs, regardless of growth factor treatment (Figure 7B, *p* < 0.05). Stretched Con ASCs and LS ASCs had higher projection factors than those in static culture; however, with GF constructs, static cells had higher projection factors than stretched cells (Figure 7C, *p* < 0.05). The circularity of stretched ASCs was significantly lower than that of static conditions for all growth factor conditions (Figure 7D, *p* < 0.05). Both the length (Figure 7E) and area (Figure 7F) of cells in stretched samples were higher than their statically cultured counterparts (*p* < 0.05). ASCs from all growth factor treatments were more aligned when the constructs were stretched (Figure 7G, *p* < 0.05).

### 3.7. Smooth Muscle-Associated Protein Expression by ASCs Cultured with Growth Factor Treatments and Mechanical Stretch

**Figure 8:**
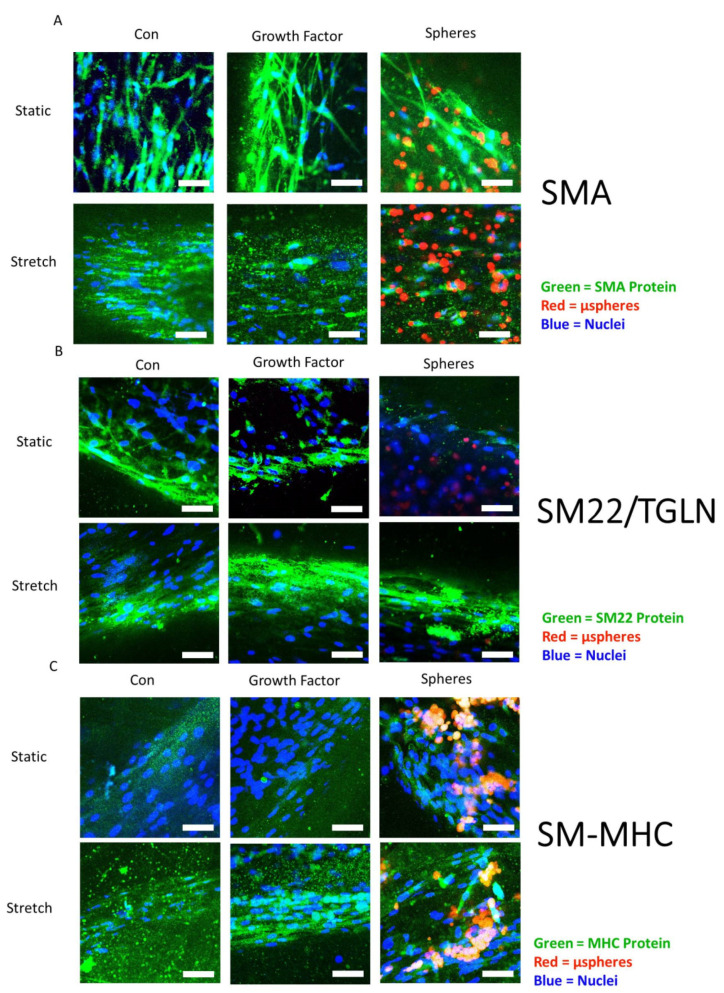
Representative images depicting the protein expression of SMA, SM22/TGLN, and MHC of ASCs. ASCs were cultured without growth factors (Con), with growth factors in the media (GF), or with growth-factor-loaded μspheres to deliver PDGF-AB and TGF-β1 (LS) under static conditions or with cyclic stretch applied for 3 days. Samples were stained on D7 for SMA (**A**), SM22/TGLN protein (**B**), or smooth muscle MHC (SM-MHC) protein (**C**) (all green). Nuclei were stained with DAPI (blue). Loaded μspheres can be seen in some images in red in the LS groups. The scale bars represent 50 μm.

**Figure 9:**
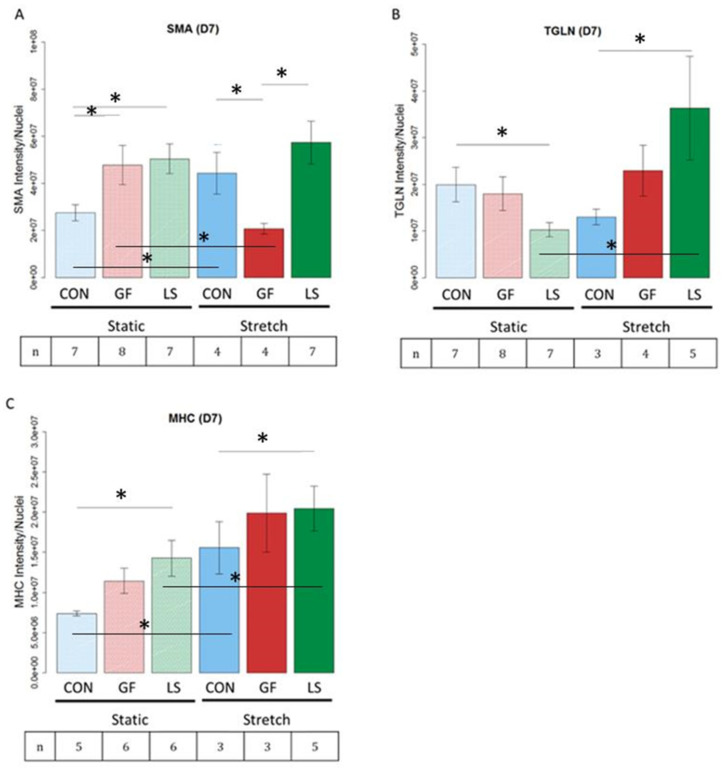
Protein expression of smooth muscle protein from ASCs in mechanically stretched constructs compared to statically cultured constructs and differentiated with different growth factor delivery methods (D7). (**A**–**C**) ASCs were cultured without growth factors (CON), with growth factors in the media (GF), or with growth-factor-loaded μspheres to deliver PDGF-AB and TGF-β1 (LS) under static conditions or with cyclic stretch applied for 3 days. Lines ending above conditions with an asterisk above indicate statistical differences between cells with different groups (*p* < 0.05). The number of biological replicates is depicted in the box under each treatment group.

Hydrogels were stained for smooth muscle actin (SMA), transgelin (TGLN or SM22), and smooth muscle myosin heavy chain (MHC), as shown in the representative images in Figure 8. These proteins were chosen as indicators of early, intermediate, and late differentiation [2], respectively. SMA and MHC are particularly important for force production while TGLN modulates the contractile apparatus. Figure 9 presents quantitative protein expression data from the same panel of markers stained in Figure 7. Under static conditions, LS ASCs treated with TGF-β1 and PDGF-AB expressed the highest amounts of SMA and smooth muscle MHC. In LS ASCs, the expression of SMA (Figure 9A, *p* < 0.05) and smooth muscle MHC (Figure 9C, *p* < 0.05) was significantly higher than in control-treated ASCs but statistically not different from the SMA and MHC expression of GF-treated ASCs (i.e., unloaded µspheres with GF in the media). However, under static conditions, TGLN protein expression was significantly lower in LS-treated vs. control hydrogels (Figure 9B, *p* < 0.05). Stretching the constructs altered this response. After 3 days of cyclic stretch, LS-containing hydrogels exhibited the highest amounts of SMA, TGLN, and smooth muscle MHC expression. Statistically, LS hydrogels contained significantly increased SMA compared to the GF group (Figure 9A, *p* < 0.05) and significantly higher TGLN (Figure 9B, *p* < 0.05) and MHC (Figure 9C, *p* < 0.05) expression compared to control hydrogels. Interestingly, GF ASCs that were stretched produced significantly less SMA than ASCs in control hydrogels when also stretched (Figure 9A, *p* < 0.05).

The combination of LS and cyclic stretch led to significantly higher expression of both TGLN (Figure 9B, *p* < 0.05) and MHC (Figure 9C, *p* < 0.05), compared to LS and non-stretched (static) hydrogels. Moreover, while cyclic stretch alone significantly increased MHC compared to static conditions in the control hydrogels, the addition of LS to both the non-stretched and stretched hydrogels resulted in a significantly higher expression of the late smooth muscle MHC marker (Figure 9C, *p* < 0.05). Collectively, these data demonstrate that the combination of cyclic stretch and TGF-β1 and PDGF-AB release over time from loaded μspheres significantly improved the differentiation of ASCs, as quantified by the protein expression of intermediate and late differentiation markers.

### 3.8. Correlations between Cell Morphology and SMC Marker Protein Expression

**Figure 10:**
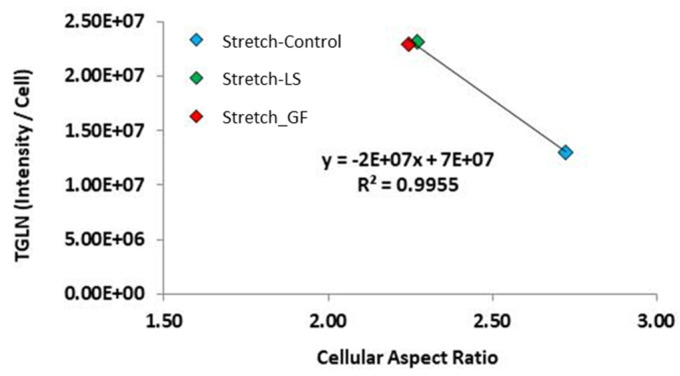
Correlation between the expression of TGLN and cell aspect ratio under stretch. For each individual experiment, the major and minor axis of each cell was measured and the aspect ratio was calculated on day 7. For each individual cell that was recorded on day 7, the *n* # of MSCs recorded is found in Figure 7H. On the *y*-axis, a single data point represents the average TGLN protein expression of all adherent cells in *n* = 3–7 individual experiments.

To determine how the modification of cell shape is related to cell differentiation, a correlation analysis was performed to determine if shape descriptors correlated with the expression of SMC marker proteins. In stretched cells, the expression of TGLN significantly correlated with the aspect ratio (Figure 10, *p* < 0.05, correlation coefficient −0.998). Moreover, under stretch, regardless of whether the growth factor delivery was through the media or from the loaded microspheres, they showed comparable values that were significantly higher than the stretched control group, which is shown in more detail in Figure 7B). Thus, further supporting that stretch modifies the MSC morphology and protein expression towards SMC myogenic differentiation.

## 4. Discussion

This study examined the separate and combined effects of growth factor stimulation and uniaxial cyclic stretch on the differentiated state of human ASCs. In particular, we expected that (i) the sustained release of PDGF-AB and TGF-β1 growth factors from μspheres can be used to induce the differentiation of ASCs into SMCs, and (ii) that a higher expression of SMC markers and a more SMC-like cell morphology can be achieved by combining growth-factor-loaded μspheres with uniaxial cyclic stretch. This study demonstrated that the combination of cyclic stretch and PDGF-AB and TGF-β1 release over time from μspheres significantly improved the differentiation of ASCs, as quantified by protein expression of early and late differentiation SMC markers, namely SMA, TGLN and smooth muscle MHC. In conjunction with these data, the delivery of growth factors via loaded μspheres produced large ASCs with a spindle-shaped, elongated SMC-like morphology. Moreover, cyclic strain produced the largest, longest, and most spindle-shaped cells regardless of the presence or absence of growth factors or the growth factor delivery method. Collectively, the protein expression and cell morphology data confirmed that the sustained release of growth factors from growth-factor-loaded μspheres can be used to induce the differentiation of ASCs into SMCs and that the addition of uniaxial cyclic stretch significantly enhances the differentiation level, as quantified by SMC protein and a SMC-like elongated cell morphology.

While the crosstalk between mechanotransduction and other signaling pathways still requires investigation, these concepts and experimental setups could be applied to multiple fields, including vascular tissue engineering as well as sphincter and bladder smooth muscle tissue engineering. Worldwide, tens of thousands of patients undergo intestinal bladder replacement surgery each year, but due to the inherent shortcomings of the current clinical methods of bladder augmentation, the development of bioactive tissue-engineered bladder repair tissue remains a large focus of urological research [64]. It has been demonstrated that mechanical stimulation is a necessary condition for bladder development, since mechanical stretch regulates the in vitro survival of human bladder SMCs [41]; therefore, the results of the current study are relevant for bladder-related tissue engineering approaches, as the bladder wall exhibits plastic behavior during fast stretch or extensive deformation [65,66]. In this context, the consideration of integrating mechanical stretch into novel technical approaches to generate SMCs or SMC-based repair tissues might help in introducing new standards for methods in regenerative medicine.

We first examined the release of growth factors from μspheres as they degraded, to allow the comparison of delivery of growth factors from μspheres to standard delivery through the culture medium. We determined that collagenase produced linear degradation rates, providing a model system to correlate degradation with growth factor release. This was similar to earlier work in our group in which μspheres were developed for other applications [38]. When loading μspheres with growth factors and degrading them with collagenase, PDGF-AB released slowly and the total amount released plateaued at around 45% of the loaded growth factor. When the μspheres were loaded with TGF-β1 and degraded with collagenase, the growth factor was released more quickly and to a higher absolute concentration. Our assay measured more TGF-β1 released into the media than we loaded into the μspheres. Some of this excess signal may be caused by gelatin degrading, or it could have been due to collagenase or another enzymatic process degrading the growth factor, thereby multiplying the epitope and producing a signal during the assay. However, the data demonstrated that the μspheres released both PDGF-AB and TGF-β1 and that the maximum level of release was observed 4 h after degradation was initiated with collagenase. In accordance with the degradation data, this implied that when cells degraded 30% of the μsphere mass, the maximum amount of growth factor would be released from the μspheres. This release profile of PDGF-AB is comparable to that of VEGF using the same μsphere system (60% recovery of VEGF after 40% of the mass had been degraded) and was released faster than BMP2 (80% recovered after 100% of the mass had dissolved) [38]. This release rate can potentially be optimized by using another type or formulation of gelatin, e.g.**,** gelatin B [38] or by changing the extent of crosslinking [37,67].

When using ASCs, we found that 30% of empty μspheres were degraded by the ASCs after 9–10 days in culture. By exposing ASCs to growth factors, either applied through the media or loaded into the μspheres, 35% of the μsphere mass was degraded by D6. This suggested that the maximum release from the μspheres occurred around D6, when the μspheres were used for inducing ASC differentiation. This increased rate of degradation was likely due to growth factor-mediated upregulation of matrix metalloproteinase production by the ASCs [68]. These experiments confirmed that we could load μspheres with exogenous growth factors and that after 6 days, the μspheres would be degraded to the point of releasing the maximum amount of growth factor. It is also possible the cells produced their own growth factors in response to these factors, but it cannot be resolved from the current setup.

We next investigated whether the method of growth factor delivery promoted the differentiation of ASCs toward a SMC-like morphology. By D7, growth factors delivered by μspheres produced SMC-like cells characterized by large cells with a spindle-shaped, elongated SMC-like morphology. This is consistent with previous findings, as growth factors have been qualitatively observed to increase the size [33,34,69,70,71] and polarization [26,33,70,71] of cells. Our data also suggest that the localized and sustained delivery of growth factors through μspheres was more effective in producing this phenotype than direct growth factor delivery through media, which is consistent with a previous study using μspheres for induction of a different cell lineage [72]. The delayed polarization from the conditions with μspheres may be due to the cells’ attraction to the μspheres [37] and, thus, the cells may have needed to degrade or rearrange the μspheres before taking on an elongated and polarized morphology.

We also examined how growth factor delivery impacted expression of selected SMC protein markers including myosin heavy chain (MHC), a late SMC differentiation marker. It is well known that growth factors used in differentiation media increase the expression of smooth muscle genes [28,29,33,34,69,71,73] but these effects are frequently investigated only for early-stage SMC markers (e.g., SMA) and intermediate-stage SMC markers (e.g., TGLN/SM22) in the differentiation of SMC-like cells derived from ASCs [74]. SMA is known to contribute to the phenotypic regulation of cells through the cytoskeleton and the signaling processes that originate from or converge on the cytoskeleton, which could regulate cell shape and differentiation [75,76]; however, SMA is not exclusive to SMCs [75]. Moreover, the expression of SMA and TGLN/SM22 often only indicates early progression towards a SMC lineage. Smooth muscle MHC is a highly specific late-stage SMC marker expressed in functionally mature SMCs [74]. Our data demonstrate that the use of μspheres loaded with PDGF-AB and TGF-β1 led to a significant (almost 2-fold) increase in smooth muscle MHC protein expression, which indicated ACS differentiation towards mature SMCs. These results were corroborated by the observed changes in ASC morphology. The protein expression of SMA and MHC was highest when the loaded μspheres were used; however, the expression of these proteins was lower in the group with unloaded μspheres and growth factors in the media. This difference engenders the question of why the application of growth factors via the culture media in the presence of unloaded μspheres vs. growth-factor-loaded μspheres led to differences in both SMC marker expression and cell morphology. It is possible that the μspheres themselves attracted the cells [37], and served as cell adhesion points, which in turn could impact the resulting cell morphology and subsequent protein expression, alignment, and differentiation [54,77]. In most cases, the two stimuli complimented one another and promoted differentiation as is evident in much of the data presented here.

It is important to note that, in addition to being expressed in SMCs, SMA and, to a lower extent, TGLN, are known to be expressed in myofibroblasts [78,79,80]. Moreover, the conditions that we used in this study could theoretically induce some degree of differentiation of ASCs into myofibroblasts. For example, TGF-β1 induces the differentiation of ASCs into myofibroblasts [81] by activation of the mechanosensitive Rho–actin–megakaryoblastic leukemia 1 (MKL1) transcriptional pathway [82] and strain and/or stiffening of the ECM can liberate latent TGF-β1 present in the ECM [83,84] via integrin-dependent mechanically induced mechanisms [85]. While we show that we achieved the highest expression of TGLN as well as smooth muscle MHC, which is absent in myofibroblasts [79,80], using stretch and growth-factor-loaded μspheres, the cells obtained after treatment may not be a pure population of SMCs but may contain some myofibroblasts. A quantification of the percentage of myofibroblasts vs. SMCs by double labeling the cells with SMA and smooth muscle MHC and quantification of double positive (SMC) vs. single positive (myofibroblast) cells by cell sorting should be considered in future studies to help determine the percentage of ASCs that were differentiated into SMCs.

Our final objective was to examine how cyclic strain in combination with μspheres influenced the differentiation of ASCs into SMCs. Stretch has been documented to inhibit the synthetic SMC phenotype and induce a more contractile phenotype [51]. Although rarely quantified, other groups observed that cyclic stretch qualitatively increases cell length [42,49,50,51,86], area [42,86], polarization [20,47,50] and quantitatively increases cell alignment [20,42,47,48,49,50,51]. We found that stretch enhanced the size, polarity, spreading, and alignment of ASCs into a more SMC-like morphology regardless of which growth factor treatment they received. In the present study, we showed that, in all experimental conditions, under 3D conditions, stretch significantly increased the protein expression of TGLN in ASCs. Moreover, under stretch conditions, the expression of TGLN correlated with an increase in cellular aspect ratio, which is representative of the ratio of the cells’ width to height. In our previous two studies using bone marrow-derived MSCs, stretch similarly increased TGLN expression [46,61]. When comparing stretch vs. no stretch, TGLN expression correlated with cell solidity when bone marrow MSCs were plated onto compacted 2D collagen hydrogels [61]. This collectively suggests that the expression of TGLN is responsive to stretch and associates with changes in cell geometry, consistent with cell elongation that is generally described in SMC myogenic differentiation [40,48,53,57]. Together these studies confirm that stretch modifies MSC morphology and protein expression towards SMC myogenic differentiation.

Moreover, in regard to stretch, regardless of the presence or absence of growth factors or the growth factor delivery method, cyclic strain produced the largest, longest, and most spindle-shaped SMC-like cells. Additionally, after the application of 3 days of cyclic stretch, ASCs in hydrogels with loaded μspheres exhibited the highest amounts of SMA, TGLN, and smooth muscle MHC expression. Statistically, cyclic stretch in combination with loaded μspheres led to more SMA but comparable TGLN and MHC protein expression, compared to stretched cells with growth factor delivered via media. These data suggest that the mechanism of growth factor delivery may not matter when combined with cyclic stretch. However, the shape data suggest a more straightforward effect: that growth-factor-loaded μspheres offer a slight edge over the soluble analog. In nearly every case, cyclic stretch increased SMC protein expression in ASCs over their analogous controls, and the addition of loaded μspheres resulted in a higher expression of the SMC differentiation markers. Collectively, the combination of cyclic stretch, and TFG-β1 and PDGF-AB release from loaded μspheres significantly improved the differentiation of ASCs, as quantified by altered cell morphology and protein expression of intermediate and late differentiation markers. The application of these techniques to smooth muscle tissue engineering not only improves our technical ability to generate SMCs, but also confirms that quantitative cell morphology can be used as an additional ASC differentiation phenotypic marker.

## Data Availability

The datasets used and/or analyzed during the current study are available from one of the corresponding authors upon reasonable request.
